# Variation in decomposition stages and carrion insect succession in a dry tropical climate and its effect on estimating postmortem interval

**DOI:** 10.1080/20961790.2020.1733830

**Published:** 2020-04-09

**Authors:** Kirsty Griffiths, Matt N. Krosch, Kirsty Wright

**Affiliations:** a Scientific Section, Forensic Services Group, Queensland Police Service, Brisbane, Queensland, Australia; b Quality Management Section, Forensic Services Group, Queensland Police Service, Brisbane, Queensland, Australia; c Genomics Research Centre, Institute of Health and Biomedical Innovation, Queensland University of Technology, Brisbane, Queensland, Australia

**Keywords:** Forensic sciences, forensic entomology, postmortem interval, Diptera, Coleoptera, decomposition

## Abstract

Insects have an important role in minimum postmortem interval (PMI_min_) estimation. An accurate PMI_min_ estimation relies on a comprehensive study of the development and succession of local carrion insects. No published research on carrion insect succession exists for tropical north Queensland. To address this, we aimed to obtain preliminary observational data concerning the rate of decomposition and insect succession on pig carcasses in Townsville and compare these with other regions of Australia and overseas. Adult insects were collected daily from three pig carcasses for 30 d during summer and identified to family level. Observations of decomposition rate were made each day and progression through the stages of decomposition were recorded. Adult insects were identified to family and their presence/absence used as a proxy for arrival at/departure from the remains, respectively. These preliminary data highlight several interesting trends that may be informative for forensic PMI_min_ estimation. Decomposition was rapid: all carcasses were at the dry/remains stage by Day 5, which was substantially quicker than all other regions in the comparison. Differences were also observed in the presence/absence of insect families and their arrival and departure times. Given the rapid progression through early decomposition, we argue that later-arriving coleopteran taxa may be more forensically informative in tropical Australia, in contrast with temperate regions where Diptera appear most useful. This research contributes preliminary observational data to understanding insect succession patterns in tropical Australia and demonstrates the critical need for comprehensive local succession data for each climatic region of Australia to enable accurate PMI_min_ estimation. These data will inform future research targeted at gaining a more comprehensive understanding of insect succession in the Australian tropics.

**Key points:**
- We obtained preliminary observational data concerning the rate of decomposition and insect succession on pig carcasses in tropical Australia.
- Decomposition was rapid: all carcasses were at the dry/remains stage by Day 5.
- Coleopteran taxa may be more forensically informative in tropical Australia than dipterans.

## Introduction

Postmortem interval (PMI) is the time between death and the discovery of the body. An accurate PMI is vital in the investigation of a suspicious death or homicide. Once an accurate estimation is achieved it can assist in identification of the deceased and targeting of suspects. Inaccurate PMIs can seriously impact an investigation by falsely eliminating suspects, contradicting witness accounts, increasing the time taken to retrieve other evidence, and creating inconsistencies with other evidence which may impact on judicial outcomes.

The classification of decomposition into distinct stages is subjective, but the most widely recognised classification was described by Payne [1]. The six stages of decomposition he first described are: fresh, bloated, active decay, advanced decay, dry stage and remains [1]. However, the distinction between the final two stages can be very difficult, and these two stages are commonly merged together into dry/remains [2,3]. The rate of decomposition is influenced by both intrinsic and extrinsic factors. Intrinsic factors include age and body composition, cause of death, and integrity of the body [4]. Extrinsic factors include temperature, degree of insolation, humidity, precipitation, season, geography, habitat, scavengers and access to the body [4–6]. In tropical regions, the early stages of decomposition can proceed rapidly, with bodies reaching advanced decay in a matter of days [7].

Insects are useful for succession studies because they are the first to detect and find remains, are present at all stages of decomposition, and can be specific for certain regions or seasons [8–10]. Forensic entomologists can make an estimation of minimum PMI (PMI_min_) by analysing the development and/or succession of insects located on human remains [10]. PMI_min_ is determined by estimating the minimum period of insect activity on the decomposing body. Using either of these methods (development or succession) requires basic reference data from experimental studies to develop a model of arthropod development and succession on carcasses [11]. Forensic entomological estimation of PMI_min_ is therefore founded upon comprehensive knowledge of both associations of insect taxa with a certain stage(s) of decomposition and of the life cycle of these taxa [12].

There are two orders of insects that are of particular forensic importance: Diptera (true flies) and Coleoptera (beetles) [4,5]. These two major insect groups are notable for their activity and frequency of occurrence on human remains. Diptera tend to have a peak abundance at the earlier stages of decomposition, whereas Coleoptera generally colonise remains during the later stages [8]. In situations where the PMI_min_ is greater than 48 h, the forensic entomologist must consider succession patterns of the entire carrion insect community, including familiarity with local arthropod communities and a comprehensive knowledge of succession patterns based on local experimental studies [12,13].

There is a lack of published succession data for carrion insects for the Australian tropics or dry tropics, with the bulk of such research conducted in temperate and subtropical regions [14–21]. Accordingly, there are no published models of insect succession available that are considered reliable for PMI_min_ estimation for tropical north Queensland. Furthermore, because decomposition in the tropics can proceed rapidly, and thus the utility of the well-known dipteran is reduced, there is a need to understand total insect succession patterns up to the dry/remains stage to encompass insect groups that may be more informative for longer PMIs. To address this, the objective of this project was to obtain preliminary data concerning stages and rate of carrion decomposition and associated insect succession patterns in Townsville, north Queensland. The preliminary insect succession data were qualitatively compared with those for temperate regions of Australia and elsewhere globally to determine if there were any substantial differences that could affect PMI_min_ estimation between geographical areas and climactic zones.

## Materials and methods

**Figure 1.**
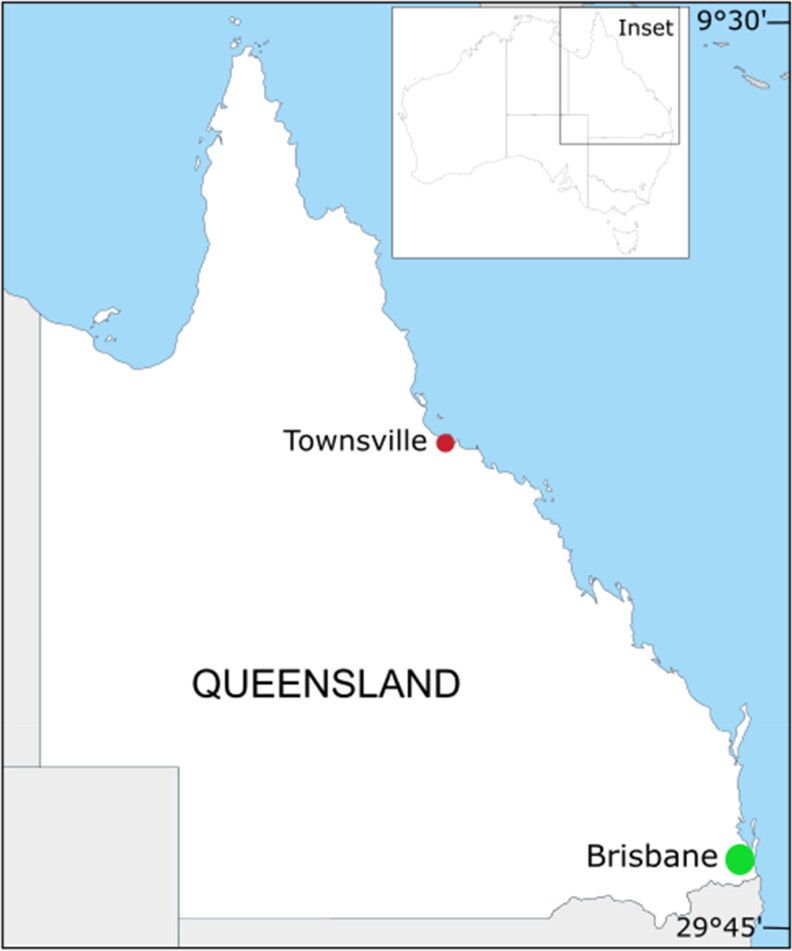
Geographical location of study region (Townsville) in tropical north Queensland, Australia.

**Figure 2.**
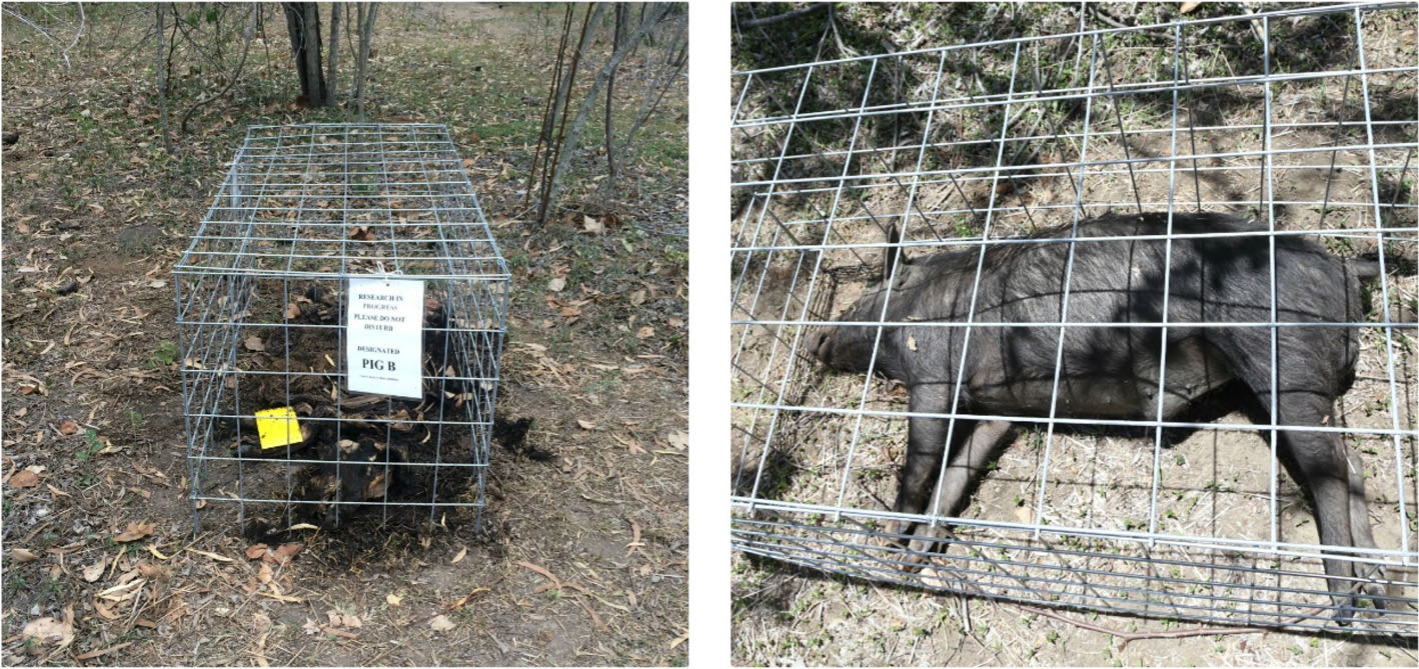
Example of the steel mesh cages used to protect the pigs from predation and scavengers.

Three wild female pigs weighing 37 kg (A), 35 kg (B) and 45 kg (C — control) were obtained from the Burdekin Shire Council as part of their annual aerial cull. The animals were killed by firearm with all sustaining a wound to the torso (consistent across all three animals) and were immediately transported to the study site in Townsville (19.42°S, 146.95°E, 19 m above sea level—Figure 1). We acknowledge here that wild pigs may possess different body tissue characteristics to domestic pigs; however, we believe they can still be instructional for decomposition studies [22]. All pigs used were mid-brown in skin tone and only lightly–moderately hairy. We also note the body mass variation between pigs; however, as they all fell within the medium/large size bracket of Matuszewski et al. [23] (35–50 kg), we believe this variation had little influence on the results. The research site was situated on a private rural property adjacent to a protected area of native bushland. The Townsville region has a dry tropical climate with distinct wet and dry seasons. This study was conducted during summer 2015–2016, commencing on 16th December 2015. The average daily temperature range is 24 °C–31 °C in summer with over 13 h of daylight per day (Australian Government Bureau of Meteorology, www.bom.gov.au). Carcasses were placed in the same orientation 20 m apart, directly on the ground on loose dirt, grass and leaf litter, near bushes in partial shade, and covered with a steel mesh cage to prevent scavenging from vertebrates (Figure 2).

The first examination was conducted on Day 2 (between 2 pm and 6 pm) and then daily up to 30 d at approximately the same time of day. Insects were collected from two of the pigs (A and B), while the control pig was left untouched to show that insect collection did not affect the rate of decomposition. The state of the carcass and stage of decomposition were documented with written reports and photographs. Definitions of decomposition stages were based on published literature [1].

Flying insects were first collected with a sweep net using four standard sweeps above the carcass. Sticky traps were laid and left overnight to collect nocturnal visiting insects, and a fresh trap was used to collect flying insects during the examination period. As time progressed and the number of flying insects reduced, the number of standard sweeps increased to collect insects that were clearly present but were not captured in the first four sweeps. Insects present on, around, and under the carcass were collected manually by searching for 20 min. The head and legs of the carcass were lifted to sample underneath. All insects were preserved in a solution of 70% ethanol and stored for identification. Qualitative assessment of insect abundance (in addition to recording presence/absence) was made during each collection such that discussion of changes to relative abundance of each taxon across decomposition stages could be made.

Daily temperature was recorded using a data logger (Thermodata, Brisbane, Australia) and rainfall was monitored by a rain gauge positioned close to the ground on the cage of the control carcass. Daily weather data, including humidity, were also collected from the Australian Government Bureau of Meteorology. Insect samples were sorted and identified to family level. Only insects of recognised forensic relevance were used for comparison purposes. Insect identifications were determined using publicly available taxonomic keys for identification [24–27]. Only adult insects were collected and identified for this study; larvae were collected but not identified or included as part of this research. We recognise that by only including adult insect data we may have inadvertently excluded taxa that were present but collected only as immatures; however, we do not expect this to have significantly impacted our analysis. Similarly, it is possible that rare taxa may have been missed during sampling, but this problem plagues all studies in this field and was ameliorated by appropriate and judicious sampling effort. When completing insect succession timelines, an insect was classified as present whether it was found on one pig or both (A or B). The arrival day was classified as the arrival of the first member of a given insect family, and the departure day was classified as the day the last member of that family was observed.

Decomposition stage and insect succession data were compared with selected previous studies from elsewhere in Australia (southeast Queensland [16], coastal New South Wales (NSW) [3], Victoria [14], and southwest Western Australia (WA) [21—the 2003 data for unclothed carcasses]), and globally (Campinas, Brazil [28], Buenos Aires, Argentina [29], and Saskatchewan, Canada [30]). These locations experience different climatic conditions to Townsville, from subtropical (southeast Queensland) and warm temperate (Campinas), to cool temperate (Victoria, NSW, southwest WA, Buenos Aires). The Victorian study lacked data for the decomposition stage and was only included for comparison of insect succession, whereas the Brazilian and Argentinian data were only included for decomposition stage comparison. The included studies were generally conducted during summer (except the WA (unclothed 2003 data) and Brazilian studies in autumn) and for durations ranging from 28–98 d. All studies reached the dry/remains stage.

Comparisons of insect succession data were made based only on the most forensically significant insect families: Calliphoridae, Sarcophagidae, Muscidae, Piophilidae, Histeridae, Dermestidae, Staphylinidae, Cleridae, Trogidae, Silphidae and Scarabaeidae. We excluded any data that related to only immature stages (e.g. Victoria) to ensure direct comparison to the adult insect collection data from this study. For studies where identifications were made to lower classifications, all species within each family were grouped together. Succession graphs were made for Townsville, NSW, WA, Victoria, and Canada. These regions were chosen for comparison as they were the only published research available with data comprehensive enough to allow for meaningful comparison. For the purposes of this research, a meaningful difference is considered plus or minus (+/−) 1 d, to avoid errors in police investigations.

## Results

### Stages and rate of decomposition

**Figure 3.**
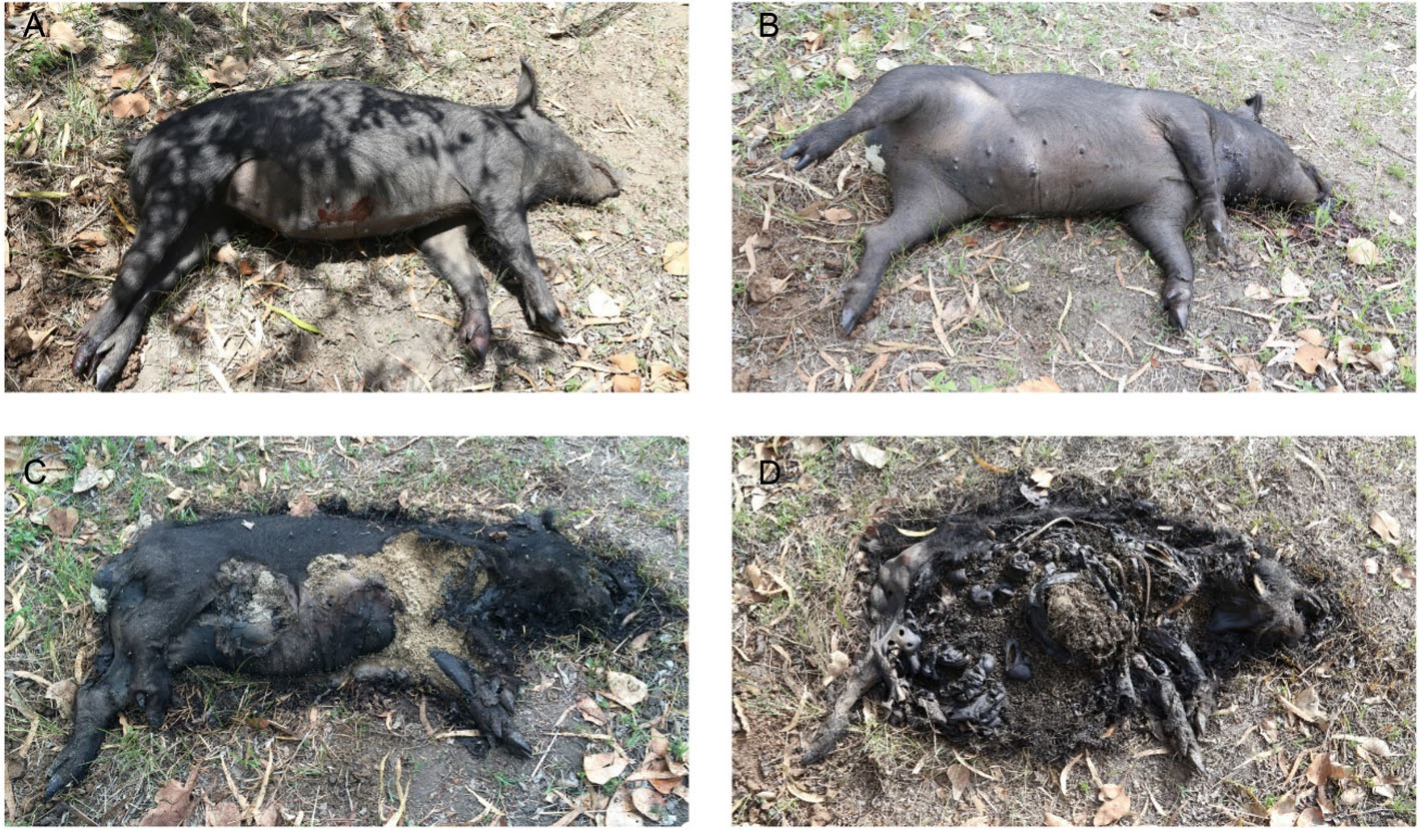
Stages of decomposition as observed on the control pig in this study. (A) Day 1—fresh; (B) Day 2—bloated; (C) Day 3—active decay; (D) Day 4—advanced decay.

**Figure 4.**
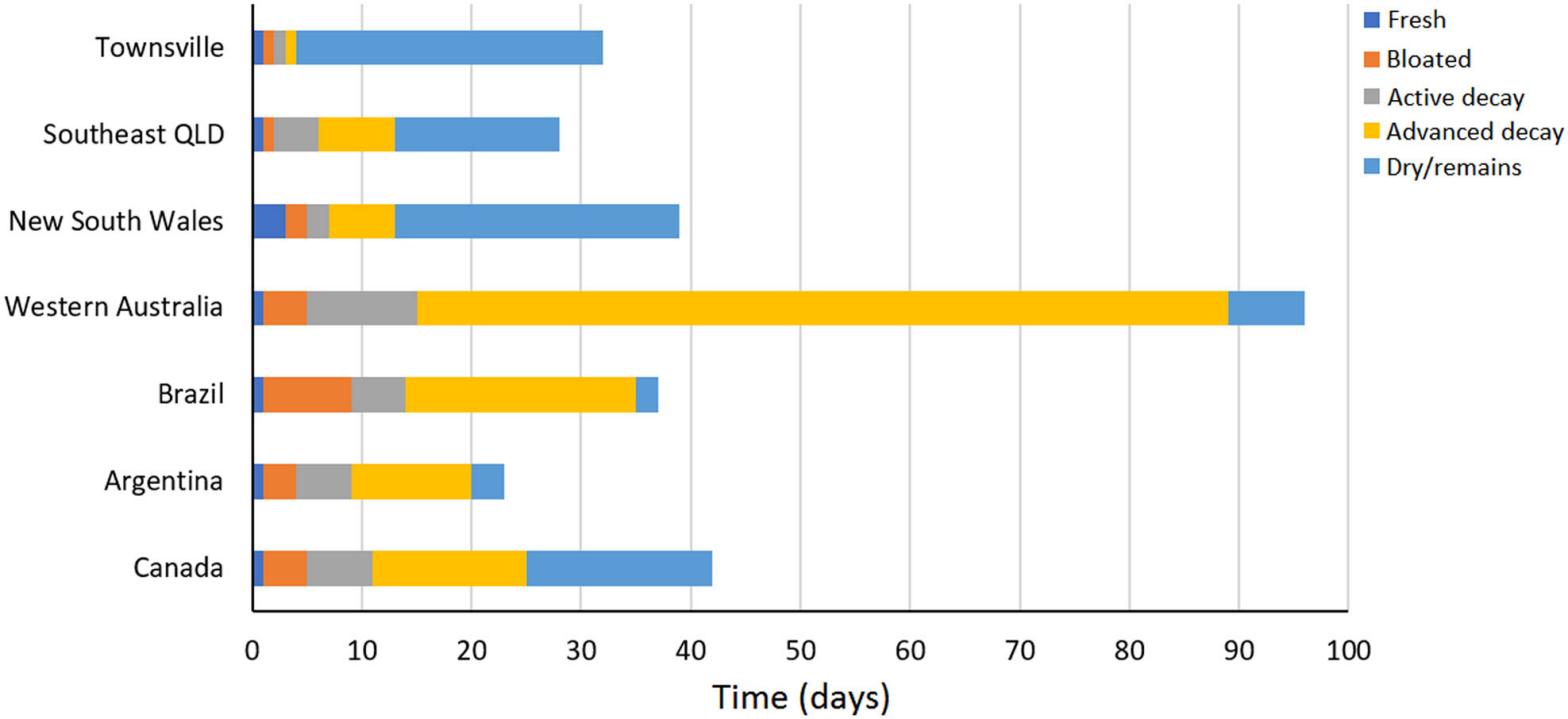
Comparison of published durations of decomposition stages between regions, data from Table 1. QLD: Queensland.

The length of each decomposition stage from this research was compared with published data to explore differences (+/− days) among regions within Australia and overseas (Table 1). For Townsville, the carcasses were all fresh at Day 1, bloated at Day 2, at the active stage on Day 3. Pigs A and B were at the advanced stage on Day 4 while the control pig reached this stage on Day 5. The pigs were all at the dry/remains stage by at least Day 5 (Figure 3). Compared with data from other regions, the observed decomposition was exceedingly rapid, occurring more quickly than any other region by 2 to 3 d for fresh, 1 to 7 d for bloated, 1 to 8 d for active decay, and 5 to 20 d for advanced decay (Table 1, Figure 4).

**Table 1.** Duration of each decomposition stage by region (days). Dry/remains durations are recorded until the end of the respective study period. The difference in fresh, bloated, active and advanced decay stage durations reported in previous studies and compared with this study is shown in parentheses.

Stage	Townsville	Southeast QLD [16]	NSW [3]	Southwest WA [21]	Brazil [28]	Argentina [29]	Canada [30]
Fresh	1	1	3 (+2)	4 (+3)	1	1	1
Bloated	1	1	2 (+1)	3 (+2)	8 (+7)	3 (+2)	4 (+3)
Active decay	1	4 (+3)	2 (+1)	9 (+8)	5 (+4)	5 (+4)	6 (+5)
Advanced decay	1	7 (+6)	6 (+5)	12 (+11)	21 (+20)	11 (+10)	14 (+13)
Dry/remains	26	15	26	NA	2	3	17

+/− days indicates difference from Townsville for each stage. QLD: Queensland; NSW: New South Wales; WA: Western Australia; NA: not available.

### Insect succession

**Figure 5.**
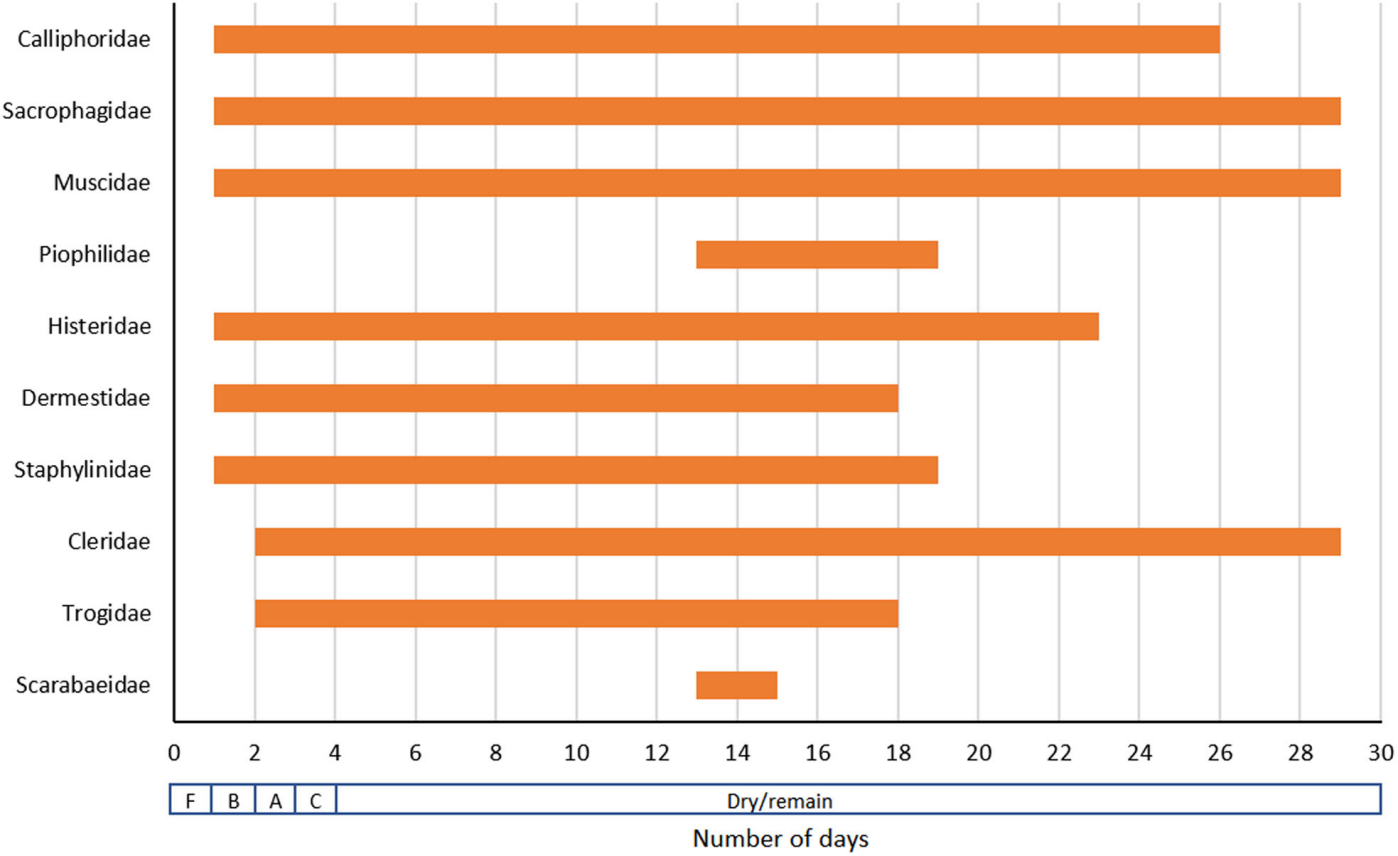
Insect succession data recorded from pig carcasses in this study. Stages of decomposition are indicated below the *x*-axis (F: fresh, B: bloated, A: active decay, C: advanced decay).

Insect succession results for Pig A and Pig B were not dramatically different, suggesting they are true replicates (Table 2, Figure 5). No difference was observed between the control carcass and those that insects were collected from, thus implying that insect removal had no impact on decomposition or insect succession. Because the carcasses transitioned rapidly from fresh to bloated to active decomposition, it was difficult to attribute any particular succession of insects to each stage of decomposition. In the first 2 d of collection, the dipteran families Calliphoridae, Sarcophagidae and Muscidae were the dominant insect groups present. No representatives of the coleopteran family Silphidae were recorded, hence this group was excluded from further comparison to temperate regions. After Day 3, on all carcasses, the number of Diptera declined markedly, making collection much more difficult. After periods of rain, the number of Diptera was noted to increase again for a short period of time and decrease again after a day of no rain.

**Table 2.** Insect succession data recorded from two pig carcasses in an open grassed habitat in Townsville, tropical north Queensland.

Day of decomposition	1	2	3	4	5	6	7	8	9	10	11	12	13	14	15	16	17	18	19	20	21	22	23	24	25	26	27	28	29
Temp (°C) high	33	33.5	34	34	32.5	34	34	34	34	34	35	37	31	32.5	31.5	30.5	30.5	32.5	33.5	34	38.5	38.5	40	35	34.5	30.5	33	31.5	35
Temp (°C) low	24.5	22	24	23	22.5	24.5	23.5	23	23	22.5	24.5	24	23.5	22.5	24	22.5	21.5	21	23.5	23.5	23.5	23.5	19.5	23	24	24.5	22.5	20.5	20.5
Relative humidity (%)	60	53	60	54	52	58	57	57	59	64	65	62	88	91	57	59	60	65	71	66	48	26	47	66	66	91	61	57	61
Rain (mm)	4	0	0	0	0	0	0	0	0	0	0	55	40	15	5	0	0	15	7	0	0	0	0	0	0	0	0	0	0
Insect family	Number of trial pig carcasses insect family present on																												
Calliphoridae	2	2	2	2	1	1		2	2	1	2	2	2	2	1	1	2	2	2	1		1				1			
Sarcophagidae	2	2	2	1	2	2	1	2	2	2	2	2	2	2	2	2	1	2	2	2	2	2	2	2	2	2	2	2	2
Muscidae	2	2	2	2	1	2	2	1	2		2	2	2	2	1	2	2		1	2	2	2	2	2	2	2	2	2	2
Piophilidae													1	1			1		1										
Histeridae	2	2	1	2	2	2	2	2	1	1	1	2			1		1						1						
Dermestidae	2	2	2	2	2	2	2	1	2	2	2	2	2	2	1	2	1	1											
Staphylinidae	1				1	1					1	2	2	2	1		1	1	2										
Cleridae		1	2	2	1	1	1	2	2	2	2	2	2	2	2	2	2	2	1	1	2	1	1	2	2	2	2	2	1
Trogidae		1					1											1											
Scarabaeidae													1	2	1														
Unidentified sp. 1																								1	1	2	1	2	2
Unidentified sp. 2																										2	1	1	1
Unidentified sp. 3 (larva)																2	2	2	2	2	2	2	2	2	2	2	2	2	2

Shaded columns represent different stages of decomposition observed in this study (dark grey: bloated, mid-grey: active decay, light grey: advanced decay, white: dry/remains).

Histeridae were present from Day 1–8 in large numbers and were still present periodically until Day 23, but at lower abundance. Cleridae were present from Day 2, but abundance varied throughout the study period, being common in the early stages of decomposition and then abundant again during the dry/remains period. Dermestidae were present at high abundance until Day 14, and present in smaller numbers until Day 18. Scarabaeidae were present for only a short time, which coincided with a period of heavy rainfall. Both Trogidae and Scarabaeidae were only ever present in very small numbers (≤3); it is possible that the removal of these specimens for examination could have affected their continued presence. Staphylinidae was represented by two morphotypes which colonised the carcass at different times and for different durations. The first type was seen for only 1 d (Day 1) on one carcass. The second type arrived on Day 5 and was present until Day 19 on both carcasses. There appeared to be some association between the presence of this family and rainfall, with none located once the carcass remained in dry decomposition after a significant period of rainfall at Day 12 to 15, and further rainfall at Day 18 to 19.

Comparison of succession data between regions (Figure 5, Supplementary Figures S1–S4) suggested there were notable differences not only in the presence/absence of insect families, but also in arrival and departure times. As expected, arrival and departure of the dipteran families Calliphoridae, Sarcophagidae and Muscidae were broadly similar across regions (arriving during the fresh stage and present until dry/remains); however, both Muscidae in NSW and Sarcophagidae in WA and Canada departed relatively early by comparison. Piophilidae were not present for the NSW study, and were present for only a short period in both Townsville (Day 13 to 19) and Canada (Day 4 to 10), whereas in Victoria they arrived on Day 6 and were present for the remainder of the study.

Differences in the arrival and departure times of Coleoptera were more clear. Histeridae arrived at the carcass during the bloated or early active decay stages in this study, NSW, WA and probably Victoria (no decomposition stage recorded), whereas they did not arrive until dry/remains in Canada. Dermestidae showed similar variation, arriving during the bloated stage at carcasses in Townsville and WA, but not until active decay in NSW and Day 11 in Victoria (likely advanced decay), and were absent entirely from the Canadian data. Staphylinidae colonised the carcasses in Townsville and NSW during the bloated stage, but did not arrive in the other regions until advanced decay (WA, Canada) and in Victoria were present from Day 6–24. In Townsville, WA, and likely Victoria, they remained until the dry/remains stage, but departed after 2 d in Canada. Cleridae arrived during active decay in Townsville and WA, but not until dry/remains in Canada (and likely advanced decay in Victoria), and remained until the end of all study periods. Significant variation was observed for Trogidae: this group colonised carcasses during active decay in Townsville, fresh stage in NSW, and later decomposition in Victoria (Day 16), all remaining until dry/remains, and were absent from WA and Canada. Scarabaeidae were only recorded from Townsville and NSW, and were present only for 2 d during the dry/remains stage in Townsville, compared with from fresh to dry/remains in NSW. These considerable differences in the arrival and departure of the Coleoptera families show that they have the potential to provide excellent data for PMI estimation, particularly during the later stages of decomposition, when local data are obtained.

## Discussion

In this study the carcasses passed through the first three stages of decomposition within 3 d. There are several factors that could have played a role in the significant transition rate of the early stages of decomposition observed. Temperature and humidity exert significant influence on both decomposition rates and dipteran development. Summer in the tropics has both a high temperature and humidity, with the temperature at this time reaching approximately 34 °C, and a relative humidity of approximately 56%. These conditions are ideal to facilitate rapid decomposition as they are optimal for both bacterial and insect development. However, because the carcasses progressed through the early decomposition stages so rapidly, the succession of insects was difficult to relate to these early stages. Future succession studies in this region should conduct examinations more frequently in the first 48 h. Rainfall appeared to have some effect on insect succession. Once the carcasses reached the dry/remains stage of decomposition, several insect groups dropped in abundance, but increased again after some significant rainfall around Day 12 to 15 (Table 2). Staphylinidae and most dipteran taxa appeared to increase in abundance and Scarabaeidae were only present after rainfall, whereas Histeridae appeared to decrease following rain. Such pulses in insect abundance attending dry carcasses following rain is generally thought to be associated with induced rehydration of any remaining tissues thereby creating additional oviposition opportunities for gravid females [31]. Overall, Coleoptera may be more informative than Diptera for PMI estimations in tropical Australia given the observed rapid progression through the early stages of decomposition. Even once the pigs had reached the dry/remains stage after only 5 d, there were clear successional changes to insect populations.

Although it is difficult to pinpoint precisely what underpins variation in decomposition rates between each of the regions included here in the absence of controlled studies, our observations do provide some confirmation that localised data are vitally important when estimating PMI. Based on the duration of decomposition stages alone (which admittedly would not be used in isolation for PMI estimations in operational cases but provides a good illustration of differences), if a body was found in Townsville in the advanced stage of decomposition, local data would suggest a PMI of only 4 d, in contrast to 7 d using southeast Queensland data, 13 d with NSW data, or 74 d with WA data. The variation in PMI estimates ranging from 3 to 70 d is an unacceptable difference and would have definite impacts on an investigation. It could be enough to dismiss potential suspects or miss possible witnesses that could be vital to an investigation. This argues strongly for increased taphonomic research in tropical regions of Australia to understand these processes better. In addition, it would be useful to make comparisons between tropical regions globally to establish whether the patterns observed here are unique or common across the tropics.

There are several sampling effects that should be noted here for their potential influence on the results of this study. Firstly, the carcasses used here all had a lethal injury to their torso which would have provided an access point for insects. However, it was noted at the time of attendance on Day 1 that rupture points in the skin of the carcasses caused by dipteran larval activity were present not only at these injury sites, but also at several other areas on the carcasses. The mode of killing used in the studies included in our comparison varied from the use of stillborn piglets [3], the use of a bolt gun intracranial shot and carotid severance [14], captive head bolt with silicone sealant [21], a high-powered bolt to the frontal portion of the skull [30], a lethal stab wound to the heart [29], or was unreported [16,28]. The mode of killing and subsequent injury could influence variation in early decomposition rates and insect colonisation. Secondly, our insect identifications were made only on the adults and to family level. We attempted to account for this by collating data from our compared studies accordingly (i.e. excluding data for larval identifications, scoring arrival/departure at the family level). Thirdly, we recognise that our sample size for the experimental component was limited by logistical constraints and therefore limits our ability to test hypotheses about differences between regions in a statistical framework. Finally, while we attempted to compare our data against studies that closely matched our experimental parameters, there were some differences. Two studies were conducted in different seasons to ours [21,28], and some used pigs with substantially different body masses [3,28–30]. This limited ability to make robust comparisons between published studies stemming from variation in experimental techniques has been recognised previously [32]. Future work in the Australian tropics should consider repeating experiments over multiple seasons, keeping in mind the general wet/dry seasonality in contrast to temperate zone thermal seasonality. Overall, however, we believe our data are sufficiently robust that general trends can be elucidated that can be instructional both for forensic scientists with limited taxonomic expertise and for informing future targeted research in tropical regions. Such future work must consider recommendations in the literature for use of standardised and accepted sampling methods and experimental design [12,32,33].

One aspect of forensic entomology in tropical Australia that requires further work is to explore comprehensively succession patterns for insect groups that are not traditionally considered of forensic importance. There were three taxa observed here (two adults and one larval type) that could not be identified, and that colonised one or both test carcasses in the second half of the experimental period. The unidentified larva arrived on Day 17 following the significant rain period, while the two unidentified adults (likely a louse and a dipteran that we could not assign to family) arrived later. These taxa may represent novel forensic indicators in tropical Australia and may be highly informative for remains in the later stages of decomposition. Alternatively, they may simply be adventitious species and not forensically relevant (particularly the putative louse). Future work should seek to identify these taxa and determine their forensic importance.

Taken together, this study has identified several important factors which should be considered when developing future succession studies aimed at generating a comprehensive database for a given region. The application of inappropriate succession data to forensic casework can lead to significant inaccuracies in PMI_min_ estimation. Although it is near impossible for succession studies to cover all possible scenarios, it is clear that attaining data for a greater number of geographical regions and death scene scenarios, using standardised methodologies, is essential to ensure constant improvement and progress in Australian (and global) forensic entomology [5,12,32,33].

## Conclusion

The observations reported here suggest that the patterns and rates of decomposition in tropical northern Australia, along with insect succession, were markedly different compared with temperate regions of Australia and overseas. The same forensically significant families were seen across most areas, but the presence/absence of insect families, along with differences in their arrival and departure times, highlighted the need for robust local succession data. If forensic entomological data from a region different to where a body is located are used, they can introduce error to PMI_min_ estimation. Even small inaccuracies can have a significant effect on a homicide investigation, representing a significant risk of causing discrepancies with the brief of evidence, which could cause delays in prosecutions or alter judicial outcomes.
